# Silencing p75NTR regulates osteogenic differentiation and angiogenesis of BMSCs to enhance bone healing in fractured rats

**DOI:** 10.1186/s13018-024-04653-8

**Published:** 2024-03-20

**Authors:** Zhifeng Wu, Yongming Yang, Ming Wang

**Affiliations:** Department of Trauma and Arthrology, First Affiliated Hospital of Shaoyang University, Shaoyang, Hunan China

**Keywords:** Fracture healing, BMSCs, p75NTR, Sortilin

## Abstract

**Background:**

Fractures heal through a process that involves angiogenesis and osteogenesis but may also lead to non-union or delayed healing. Bone marrow mesenchymal stem cells (BMSCs) have been reported to play a pivotal role in bone formation and vascular regeneration and the p75 neurotrophin receptor (p75NTR) as being an important regulator of osteogenesis. Herein, we aim to determine the potential mediation of BMSCs by p75NTR in bone healing.

**Methods:**

Rat BMSCs were identified by flow cytometry (FCM) to detect cell cycle and surface markers. Then transfection of si/oe-p75NTR was performed in BMSCs, followed by Alizarin red staining to detect osteogenic differentiation of cells, immunofluorescence double staining was performed to detect the expression of p75NTR and sortilin, co-immunoprecipitation (CO-IP) was conducted to analyze the interaction between p75NTR and sortilin, and EdU staining and cell scratch assay to assess the proliferation and migration of human umbilical vein endothelial cells (HUVECs). The expression of HIF-1α, VEGF, and apoptosis-related proteins were also detected. In addition, a rat fracture healing model was constructed, and BMSCs-si-p75NTR were injected, following which the fracture condition was observed using micro-CT imaging, and the expression of platelet/endothelial cell adhesion molecule-1 (CD31) was assessed.

**Results:**

The results showed that BMSCs were successfully isolated, p75NTR inhibited apoptosis and the osteogenic differentiation of BMSCs, while si-p75NTR led to a decrease in sortilin expression in BMSCs, increased proliferation and migration in HUVECs, and upregulation of HIF-1α and VEGF expression. In addition, an interaction was observed between p75NTR and sortilin. The knockdown of p75NTR was found to reduce the severity of fracture in rats and increase the expression of CD31 and osteogenesis-related proteins.

**Conclusion:**

Silencing p75NTR effectively modulates BMSCs to promote osteogenic differentiation and angiogenesis, offering a novel perspective for improving fracture healing.

## Introduction

Fractures are among the most prevalent injuries affecting the musculoskeletal system, often due to bone injuries and various medical conditions [1, 2]. The process of fracture healing is complex and influenced by numerous factors, including soft tissue damage, bone displacement, and the severity of trauma [3, 4]. Fracture healing delay is characterized by the absence of healing progress within three months following the injury, while fracture non-union is described as the absence of healing within 9 months [3]. Recent data indicates that nearly 25% of open long bone fractures result in either fracture non-union or delayed healing [2], and approximately 6.8% of individuals with tibial fractures experience delayed healing [5]. Importantly, non-union or delayed fracture healing may detrimentally affect musculoskeletal functions, daily life activities, and the financial well-being of patients, and in severe cases, it can even be life-threatening [6, 7]. Currently, the primary treatment for fractures includes conservative and surgical approaches, but it is worth highlighting that the non-union rate tends to be higher with conservative treatment methods [5]. Therefore, there is an urgent need to investigate efficacious strategies for promoting bone healing and deepen our understanding of fracture repair.

Physiologically, the healing of a fracture involves processes such as angiogenesis and osteogenesis, among which angiogenesis is crucial for bone formation and regeneration [8, 9]. Notably, a compromised blood supply to the site of a bone fracture can substantially impede healing and the formation of new bone tissues [10, 11]. The intricate healing of bone fractures relies on the meticulous coordination of various cell types, such as inflammatory cells and mesenchymal stem cells (MSCs) [12], and in the context of bone healing, it is important to simultaneously improve both blood vessel formation and osteogenesis. In this regard, bone marrow mesenchymal stem cells (BMSCs) have been found to be pivotal players and serve as precursor cells for both bone and cartilage. BMSCs possess the remarkable ability for self-renewal and differentiation, ultimately giving rise to various bone matrix cells [13–15]. Extensive research has shown the significant contribution of BMSCs to bone formation and the regeneration of vascular networks [16, 17]. Moreover, studies have highlighted the potential of exosomes derived from umbilical cord mesenchymal stem cells (uMSC) to accelerate fracture healing by promoting angiogenesis [17]; thus, indicating the central role of BMSCs in the intricate process of bone healing.

Neuronal innervation of bone is an emerging research topic, with nerve-based BMSCs offering promising prospects for innovative fracture healing strategies. The p75 neurotrophin receptor (p75NTR), a member of the tumor necrosis factor superfamily, acts as a receptor for growth factors, including the nerve growth factor receptor [18, 19], and regulates multiple biological pathways, such as signaling transduction, cell differentiation and apoptosis [18]. Studies have reported that p75NTR is a key regulator of osteogenesis and influences the differentiation of osteoblasts [20, 21]. Elevated p75NTR expression has been observed in late-stage cartilage injuries and fibrotic diseases [22], while in diabetes, it increases endothelial cell apoptosis and hinders angiogenesis [23]. In addition, inhibiting p75NTR expression reduces apoptosis in neurogenic-like cells derived from BMSCs [24]. Despite these findings, the role of p75NTR in mediating the influence of BMSCs on bone fracture healing remains unexplored. This study was designed to elucidate the potential involvement of p75NTR and underlying mechanisms in bone fracture healing to obtain insights that could be used as a referential basis to improve fracture non-union and delayed healing issues.

## Materials and methods

### Isolation and identification of rat BMSCs

After euthanasia, the tibias and femurs of the rats were immersed in 75% ethanol for 5 min, after which they were separated, and any muscle tissues adhering to the long bones were removed [25]. The diaphysis of both the tibia and femur was then cut open, and cells from the ends of these long bones were aseptically transferred into a pre-chilled 50 mL centrifuge tube containing a complete culture medium (comprising MSC culture medium supplemented with antibiotics and 10% FBS). The bone marrow cell suspension was obtained by centrifugation at 1500 rpm for 5 min and added into a complete culture medium comprising MSC culture medium (AW-MC036, Abiowell, China), 10% FBS (10,099,141, Gibco, USA) and antibiotics (ST488, Beyotime, China), with the entire process maintained under aseptic conditions. Cellular morphological analysis was conducted by observing and capturing images of the cells using a microscope (DSZ2000X, Cnmicro, China).

### Cell culture and grouping

Following the isolation of BMSCs in the previous step, the cells were cultured in a complete MSC culture medium, placed in a cell culture incubator maintained at 37 °C with 5% CO_2,_ and categorized into five groups: Control, si-NC, si-p75NTR, oe-NC, and oe-p75NTR. In the Control group, BMSCs were cultured under standard conditions; in the si-NC group, BMSCs were transfected with si-NC plasmid, while in the si-p75NTR group, BMSCs were transfected with si-p75NTR plasmid. In the oe-NC group, the BMSCs were transfected with the oe-NC plasmid, and in the oe-p75NTR group, BMSCs were transfected with the oe-p75NTR plasmid. These cell groups were then cultured for 24, 48, and 72 h. Additionally, BMSCs were co-cultured with human umbilical vein endothelial cells (HUVECs) and divided into four groups: Control, si-NC, si-p75NTR, and si-p75NTR + rr-sortilin. In the Control group, the cells were cultured under standard conditions; in the si-NC group, the cells were transfected with si-NC plasmid, and in the si-p75NTR group, the cells were transfected with si-p75NTR plasmid. In the si-p75NTR + rr-sortilin group, the cells were transfected with si-p75NTR plasmid and treated with recombinant rat sortilin (rr-sortilin) protein.

### Cell transfection

Eight centrifuge tubes were used, to which 95 µL of MSC basal culture medium was added. Separately, the si-NC, si-p75NTR, oe-NC, and oe-p75NTR plasmids, along with Lipofectamine 2000 (11,668,500, Invitrogen, USA), were mixed with MSC culture medium and incubated at room temperature for 5 min. The two tubes corresponding to each plasmid were combined and incubated at room temperature for 20 min. The resultant pre-mixture was evenly distributed into the transfection wells, followed by adding 800 µL of MSC basal culture medium. The cells were then incubated at 37 °C for 6 h, after which the complete culture medium was replaced. The si-NC, si-p75NTR (HG-Ri0072564), oe-NC, and oe-p75NTR plasmids were purchased from HonorGene (HG-RO0072566, China).

### Cell counting kit-8 (CCK-8) assay

The cells were seeded in a plate at a density of 5 × 10^3^ cells/well and allowed to adhere to the plate. After removing the culture medium, 100 µL/well of prepared CCK-8 medium, consisting of 10% CCK-8 reagent (NU679, Abiowell, China) and 90% complete culture medium, was added and incubated at 37 °C with 5% CO_2_. Lastly, the plates were placed in a microplate reader (MB-530, HEALES, China) to measure the optical density (OD) value at 450 nm.

### Osteogenic induction of BMSCs

A 6-well plate was prepared by adding 1 mL of 0.1% gelatin, mixing well, and allowing it to dry. After gelatin coating, BMSCs were seeded into each well, and 2 mL of complete culture medium was added per well. The plate was subsequently incubated in a cell culture incubator. After removing the culture medium, 2 mL of Rat BMSCs Osteogenic Differentiation medium (RAXMX-90,021, Cyagen, China) was added to each well, and the medium was replaced at 3-day intervals as part of the osteogenic induction process.

### Alizarin red staining (ARS)

The assessment of BMSCs’ osteogenic differentiation was performed using ARS staining. After 2–4 weeks of induction, the culture medium was aspirated, and the cells were washed with phosphate buffer saline (PBS). Then, the cells were fixed with a 4% paraformaldehyde solution (AWI0056b, Abiowell, China) and rinsed with ultrapure water. Next, 1 mL of 0.2% ARS solution (AWI0292a, Abiowell, China) was added to each well, and the plate was gently shaken on a shaker to facilitate staining. Lastly, the cells were examined and captured under a microscope (DSZ2000X, Cnmicro, China) for observation and photography purposes.

### Adipogenic induction of BMSCs

The cells were seeded onto plates pre-coated with 0.1% gelatin and cultured in 2 mL of complete culture medium per well. The culture medium was then replaced with 2 mL of Rat Bone Marrow Mesenchymal Stem Cell Adipogenic Induction Differentiation Medium A (RAXMX-90,031, Cyagen, China) for each well. After three days, Medium A was discarded and replaced with an equal volume of Medium B, which was removed after one day, and subsequently, Medium A and Medium B were alternated in the subsequent culture process.

### Oil Red O staining

Here, Oil Red O staining was performed to evaluate the adipogenic differentiation of BMSCs. After 16 days of inducing adipogenesis, the culture medium was aspirated, the cells were rinsed with PBS, fixed using a 4% paraformaldehyde solution, and stained with a pre-prepared Oil Red O working solution (G1262, Solarbio, China) at room temperature. After staining, the cells were washed with PBS to remove any excess staining solution. Lastly, each well was filled with PBS, and the plate was examined under a microscope (DSZ2000X, Cnmicro, China) to assess the effectiveness of adipogenic staining.

### Cell cycle analysis

Flow cytometry (FCM) was performed to assess the cell cycle. Initially, the collected BMSCs and HUVECs were resuspended separately in PBS to separate them into individual cells, which were then fixed with ethanol and subsequently washed with PBS to eliminate residual ethanol. PI working solution (MB2920, MeilunBio, China) was then added to the cells and stained in the dark at 4 °C. Lastly, a flow cytometer (A00-1102, Beckman, USA) was used for cell cycle analysis.

### Apoptosis detection in BMSCs

Cell apoptosis analysis was conducted using FCM. Briefly, BMSCs were harvested and prepared for apoptosis detection by trypsin digestion without ethylenediaminetetraacetic acid (EDTA). The cells were washed with PBS, and the supernatant was removed after centrifugation. Next, the cells were resuspended in 500 µL of Binding buffer (KGA1030, KeyGene BioTECH, China), 5 µL of Annexin V-APC was added, and the mixture was gently combined. Following this, PI was added to the cell suspension, and the entire reaction was conducted away from light. Within 1 h, FCM was performed to analyze cell apoptosis.

### Surface marker identification of BMSCs

The digested BMSCs were centrifuged, and the supernatant was discarded. The cell pellet was then resuspended in 100 µL of basal culture medium and thoroughly mixed with specific antibodies (Table 1). The mixture was incubated at room temperature away from light for 30 min, following which, the cells were washed with 1 mL of 0.5% BSA-PBS solution and centrifuged for 5 min to remove the supernatant. The cell pellet was resuspended in 150 µL of 0.5% BSA-PBS and analyzed using FCM. The specific antibodies used in the study are shown in Table 1.

**Table 1:** The information on antibody

Name	Dilution rate	Cat number	Source	Company	Country
CD29	1 µg	12-0291-82	Mouse	eBioscience	USA
CD90	0.06 µg	12-0900-81	Mouse	eBioscience	USA
CD44	0.06 µg	12-0444-82	Mouse	eBioscience	USA
CD105	1 µg	12-1057-42	Mouse	eBioscience	USA
CD34	20 µL	MA1-10205	Mouse	eBioscience	USA
CD45	0.25 µg	12-0461-82	Mouse	eBioscience	USA

### Construction of rat fracture healing model

Male SD rats (age: 5–6 weeks; weight: 200–250 g) were purchased from SJA Laboratory Animal Co., Ltd (Hunan, China). After a week of acclimation feeding, subsequent experiments were performed. To establish a rat model for bone healing, the rats were initially anesthetized via intraperitoneal injection with 40 mg/kg of pentobarbital sodium. Then, a 1.2 mm diameter bone defect was created at the midshaft of the bilateral femurs, ensuring that the defect penetrated the cortex into the marrow cavity without perforating the opposite cortex [26]. A total of 18 rats were randomly allocated into three groups (*n* = 6): Model group, Model + BMSCs-si-NC group, and Model + BMSCs-si-p75NTR group. Rats in the Model group received an intravenous injection of 0.2 mL of normal saline, those in the Model + BMSCs-si-NC group were administered a tail vein injection of 0.2 mL normal saline containing 106 BMSCs transfected with si-NC plasmid, and those in the Model + BMSCs-si-p75NTR group received a tail vein injection of 0.2 mL normal saline containing 106 BMSCs transfected with si-p75NTR plasmid [13]. These injections were administered every other day. Two weeks post-surgery, the rats were euthanized, and samples were collected for subsequent analysis. All experimental procedures and animal handling were performed with the approval of the Biomedical Research Ethics Committee of Shaoyang University (No. 2021KJKT023), in accordance with the National Institutes of Health Guide for the Care and Use of Laboratory Animals, and studies involving laboratory animals follows the ARRIVE guidelines.

### Western blot (WB)

The cells and tissues were treated using a Radio Immunoprecipitation Assay (RIPA) lysis buffer (AWB0136, Abiowell, China) following the recommended instructions, and the protein concentration was determined using a Bicinchoninic Acid (BCA) assay kit (AWB0104, Abiowell, China). Subsequently, the protein was transferred onto nitrocellulose (NC) membranes (Invitrogen, USA) through electrophoresis and electroblotting, then immersed in a 5% skimmed milk solution prepared with 1×PBST and gently agitated for 90 min for blocking. Next, the membranes were incubated overnight with the primary antibody and thoroughly washed with PBST. Subsequently, the membranes were incubated with the secondary antibody (HRP goat anti-mouse IgG (H + L) and HRP goat anti-rabbit IgG (H + L)). Lastly, the membranes were treated with enhanced chemiluminescence (ECL) Plus detection reagent (AWB0650, Abiowell, China) and visualized using a chemiluminescence imaging system (ChemiScope6100, Clinx Science, China). The internal control gene used in this study was β-actin, and details regarding the primary and secondary antibodies utilized are provided in Table 2.

**Table 2:** The information on antibody

Name	Dilution rate	Cat number	Source	Company	Country
p75NTR	1:1000	AWA01539	Mouse	Abiowell	China
sortilin	1:8000	12369-1-AP	Rabbit	Proteintech	USA
Caspase3	1:1000	AWA54285	Rabbit	Abiowell	China
BAX	1:5000	AWA00856	Mouse	Abiowell	China
BCL-2	1:2000	AWA00387	Mouse	Abiowell	China
Runx2	1:1000	AWA48303	Rabbit	Abiowell	China
osteopontin	1:600	AWA43353	Rabbit	Abiowell	China
DLX5	1:300	AWA46523	Rabbit	Abiowell	China
collagen type I	1:2000	AWA00725	Mouse	Abiowell	China
BMP2	1:1000	AWA56105	Rabbit	Abiowell	China
CD31	1:1000	AWA58016	Rabbit	Abiowell	China
HIF-1α	1:1000	AWA00942	Mouse	Abiowell	China
VEGF	1 µg/mL	ab46154	Rabbit	Abcam	UK
β-actin	1:5000	AWA8002	Mouse	Abiowell	China
HRP goat anti- mouse IgG (H + L)	1:5000	AWS0001	/	Abiowell	China
HRP goat anti- Rabbit IgG (H + L)	1:5000	AWS0002	/	Abiowell	China

### Co-immunoprecipitation (CO-IP)

BMSCs were lysed using IP lysis buffer (AWB0144, Abiowell, China), and the protein-containing supernatant was collected post-centrifugation. The protein extract was allocated into four separate tubes, to which specific antibodies were added: anti-p75NTR (55014-1-AP, 1:1000, Proteintech), anti-sortilin (12369-1-AP, 1:8000, Proteintech), or anti-rabbit IgG (SA00001-2, 1:6000, Proteintech), according to the experimental requirements. After thorough mixing, the mixtures were incubated overnight. The antibody-protein complexes were then combined with pre-treated Protein A/G agarose beads and incubated with gentle agitation at 4℃ for 2 h, followed by centrifugation. The beads, which have bonded with the antibody-protein complexes, were retained, and the supernatant was discarded after washing the beads four times with IP lysis buffer. Lastly, IP lysis buffer and 5× loading buffer were added to the bead-bound complexes, which were then mixed, denatured, and prepared for subsequent protein quantification analysis.

### Real-time quantitative polymerase chain reaction (RT-qPCR)

Total RNA extraction from BMSCs was performed using Trizol (15,596,026, Thermo, America), and cDNA was synthesized using the mRNA Reverse Transcription Kit (CW2569, CowinBio, China). Primers for the target genes were designed and synthesized by Tsingke (Beijing, China). The specific amplification was conducted using the SYBR method on a fluorescence qPCR instrument (SPL0960, Thermo, USA). β-actin was used as the internal reference, and the relative gene expression levels were calculated utilizing the 2^−ΔΔCt^ method. The primer sequences utilized are shown in Table 3.

**Table 3:** Primer sequences

Gene	Sequence	Length
p75NTR	F GGCCCAGAAGGTTGTGATGA R GGGTGGAAAGTCCACTGAGG	175bp
sortilin	F GGCCAAATGGGGATCAGACA R GCAAAAAGGAAACGTCCCCC	168bp
Runx2	F CACTGGCGCTGCAACAAGACCCT R CCGGCCCACAAATCTCAGATCGT	186bp
osteopontin	F CATCACCTCACACATGGAAAGCG R GCTCTCATCATTGGCTTTCCG	199bp
DLX5	F GCCGGAGACAGAGACTTCAC R GGGGACCCTTCTGTCAAACA	226bp
osteocalcin	F CATGAGAGCCCTCACACTCC R CGCCTGGGTCTCTTCACTAC	153bp
collagen type I	F GCAAGAACCCCGCCCGCACC R GCTCTCGCCGAACCAGACATGCC	233bp
BMP2	F AGAATAACTTGCGCACCCCA R GGACCGAATGTCCGTTCCTT	197bp
β-actin	F ACCCTGAAGTACCCCATCGAG R AGCACAGCCTGGATAGCAAC	224bp

### Immunofluorescence (IF)

The expression of p75NTR and sortilin proteins in differentiated osteoblasts was assessed through IF. BMSCs were cultured on glass coverslips, fixed using 4% paraformaldehyde, and permeabilized with 0.3% Triton X-100 at 37 °C. After rinsing with PBS, a blocking step was performed using 5% BSA. The coverslips were then incubated overnight with the p75NTR antibody (AWA01539, 1:200, Abiowell) and sortilin antibody (12369-1-AP, 1:50, Proteintech). Then, the slides were incubated with Alexa Fluor 594-conjugated Goat Anti-Rabbit IgG(H + L) antibody (1:200, AWS0006c, Abiowell) and Alexa Fluor 488-conjugated Goat Anti-Mouse IgG(H + L) antibody (1:200, AWS0003c, Abiowell) for 90 min at 37℃. Nuclear staining was conducted using a DAPI working solution (C1005, Beyotime, China). Lastly, the slides were sealed with a glycerol buffer and observed using a fluorescence microscope (BA410T, Motic, Singapore).

### 5-Ethynyl-2’-deoxyuridine (EdU)

To assess the proliferation of HUVECs, 50 µM EDU medium (C10310, RiboBio, China) was added to each well and incubated overnight. After fixation with 4% polyformaldehyde, 50 µL of 2 mg/mL glycine solution was used. Subsequently, the cells were treated with 1× Apollo staining reaction solution and incubated in the dark. Following this, a permeabilization agent and methanol were introduced to the cells and then washed. Each well was treated with 1× Hoechst 33,342 reaction solution and incubated in the dark. Lastly, images were captured using a fluorescence microscope (BA410T, Motic, China).

### Wound healing assay

The cells were seeded into a 6-well plate. A sterile pipette tip was used to generate a scratch at the center of each well, which was then washed with PBS three times and supplemented with serum-free DMEM/F12 medium. Photographs were captured at 0, 24, and 48 h after the scratch using a microscope (DSZ2000X, Cnmicro, China) in at least three distinct fields of view to assess the migration capacity of HUVECs.

### Statistical analysis

In this experiment, statistical analysis was performed using GraphPad Prism 9.0, and the data are presented as mean ± standard deviation (SD). Tests for normal distribution and homogeneity of variances were conducted, and data that satisfied both of these tests were further compared. For comparisons among multiple groups, a one-way ANOVA followed by Tukey’s post hoc test was performed. A two-way ANOVA was utilized for comparisons involving different time points and multiple groups. *P* < 0.05 was considered statistically significant.

## Results

### Isolation and identification of rat BMSCs

**Fig. 1:**
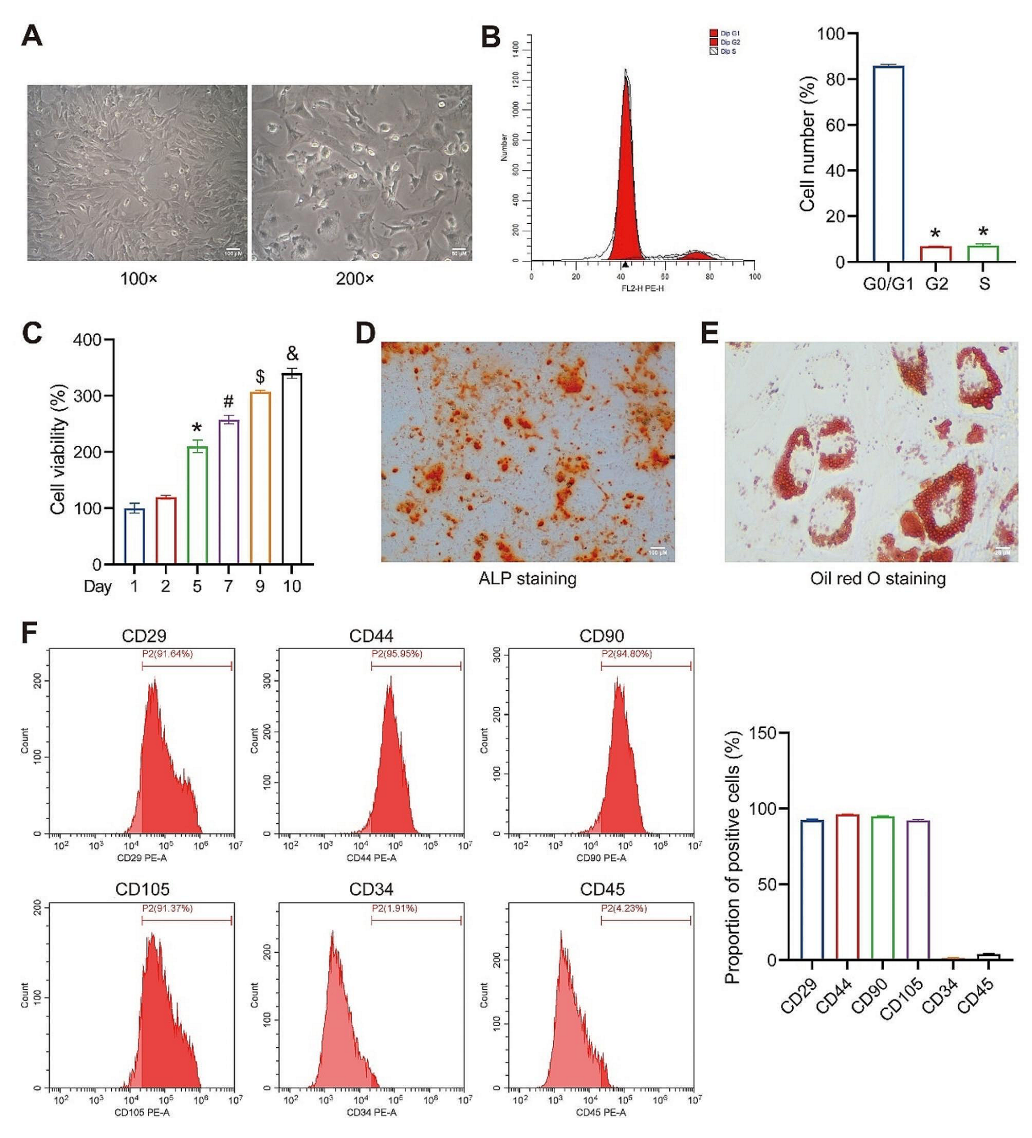
Isolation and identification of rat BMSCs. (**A**) Observation of cell morphology (Magnification: ×100, scale bar = 100 μm; ×200, scale bar = 50 μm). (**B**) FCM analysis to determine the cell cycle of the isolated cell population. *P < 0.05 vs. G0/G1. (**C**) CCK-8 analysis to determine the cell viability of the isolated cell population. *P < 0.05 vs. day 2, #*P* < 0.05 vs. day 5, $*P* < 0.05 vs. day 7, &*P* < 0.05 vs. day 9. (**D**) Observation of ARS for cell osteogenic differentiation (Magnification: ×100, scale bar = 100 μm). (**E**) Observation of Oil Red O staining for cell adipogenic differentiation (Magnification: ×400, scale bar = 25 μm). (**F**) FCM analysis of surface markers on BMSCs

Rat BMSCs were isolated from the tibia and femur and identified. Microscopic examination revealed a fibroblast-like morphology with spindle shapes and centrally positioned oval-shaped nuclei, accompanied by various cytoplasmic protrusions of varying lengths (Fig. 1A). FCM analysis indicated that most cells were in the G0/G1 phase, with only a minimal proportion in the G2 and S phases (Fig. 1B). Over time, cell viability was found to increase significantly (Fig. 1C). Subsequent osteogenic and adipogenic induction of the passaged cells resulted in the occurrence of calcium nodules and lipid droplets, as confirmed by ARS and Oil Red O staining, respectively (Fig. 1D and E). Studies have reported that CD29, CD44, CD90, CD105, and others can serve as surface markers for BMSCs [27]. Our results showed that the cell surface markers exhibited positive expression of CD29, CD44, CD90, and CD105, while CD34 and CD45 were negative (Fig. 1F), indicating the successful isolation of rat BMSCs.

### p75NTR promotes apoptosis in rat BMSCs

**Fig. 2:**
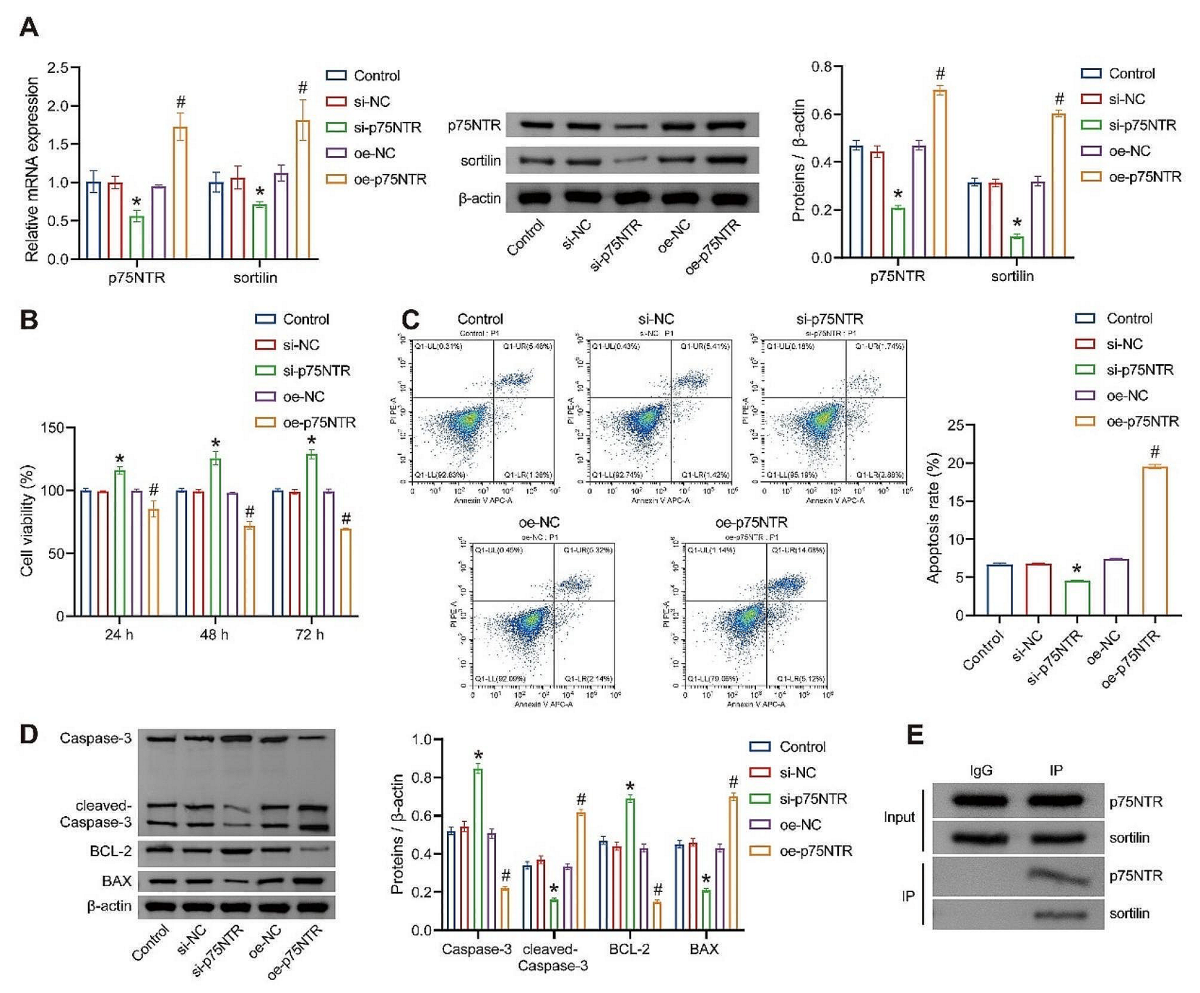
p75NTR promotes apoptosis in Rat BMSCs. (**A**) Expression of p75NTR and sortilin was detected by RT-qPCR and WB. (**B**) Cell viability assessment by CCK-8. (**C**) FCM analysis of cell apoptosis. (**D**) Detection of apoptosis-related proteins Caspase-3, BCL-2, and BAX expression by WB. (**E**) CO-IP analysis of protein-protein interaction between p75NTR and sortilin. *P < 0.05 vs. si-NC. #*P* < 0.05 vs. oe-NC

After successfully isolating BMSCs, we investigated whether p75NTR plays a role in mediating apoptosis in rat BMSCs. Our results demonstrated that transfection with si-p75NTR significantly reduced the expression of p75NTR, while transfection with oe-p75NTR significantly increased its expression, confirming the successful interference and overexpression of p75NTR. Moreover, following si-p75NTR transfection, there was a significant decrease in the levels of sortilin expression, whereas oe-p75NTR transfection led to an increase in sortilin levels (Fig. 2A). These findings preliminarily suggest that p75NTR may positively regulate the expression of sortilin in BMSCs. Subsequently, we assessed the impact of p75NTR on BMSCs’ cell viability and apoptosis levels. Si-p75NTR transfection resulted in increased cell viability and reduced apoptosis rates, while oe-p75NTR transfection led to decreased cell viability and increased apoptosis rates (Fig. 2B and C). Next, we examined the expression levels of apoptosis-related proteins. Previous studies have reported that B-cell lymphoma 2 (BCL-2) inhibits apoptosis, while BCL2-Associated X (BAX) is a pro-apoptotic member of the BCL-2 protein family [28, 29]. Both cleaved caspase-3 and BAX proteins have been implicated in promoting apoptosis [29, 30]. Our results indicated that si-p75NTR transfection significantly reduced the expression levels of cleaved-Caspase-3 and BAX while increasing Caspase-3 and BCL-2 expression. Conversely, oe-p75NTR transfection significantly elevated the expression levels of cleaved Caspase-3 and BAX while reducing Caspase-3 and BCL-2 expression (Fig. 2D). This suggests that p75NTR acts as a promoter of apoptosis in BMSCs. Furthermore, our experiments revealed an interaction between p75NTR and sortilin (Fig. 2E). Collectively, these results suggest that p75NTR and sortilin interact with each other, and p75NTR could positively regulate the expression of sortilin. Additionally, p75NTR promotes apoptosis in rat BMSCs.

### Inhibition of osteogenic differentiation of rat BMSCs by p75NTR

**Fig. 3:**
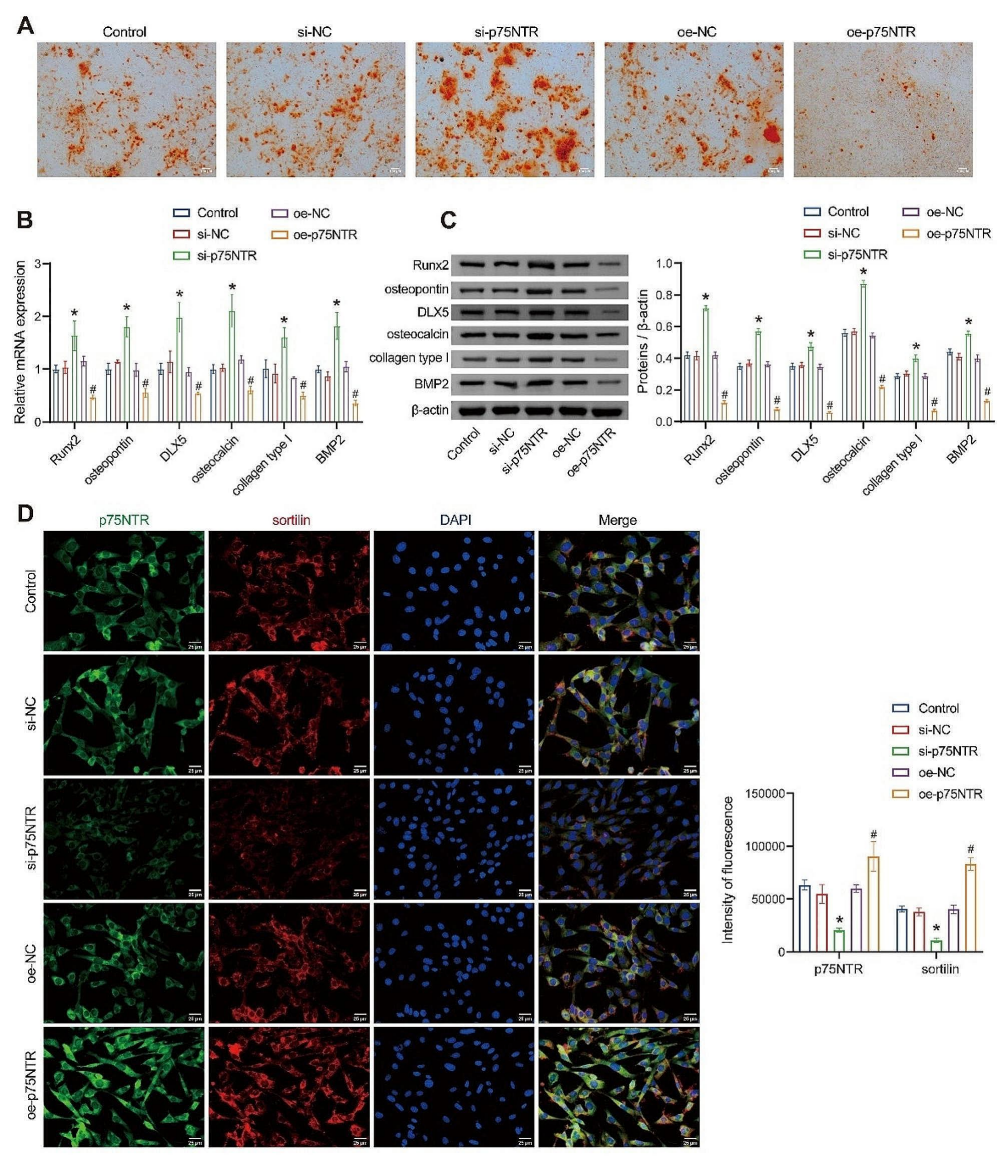
Inhibition of osteogenic differentiation of rat BMSCs by p75NTR. (**A**) Measurement of cell osteogenic differentiation ability using ARS assay (Magnification: ×100, scale bar = 100 μm). (**B**) RT-qPCR detection of gene expression related to osteogenesis. (**C**) WB analysis was used to detect the expression of genes associated with osteogenesis. (**D**) IF double-staining was performed to assess the level of p75NTR and sortilin proteins in differentiating osteoblasts (Magnification: ×400, scale bar = 25 μm). *P < 0.05 vs. si-NC. #*P* < 0.05 vs. oe-NC

After confirming the involvement of p75NTR in BMSC apoptosis, we investigated whether p75NTR also played a role in mediating the osteogenic differentiation of rat BMSCs. Initially, BMSCs underwent osteogenic induction, followed by ARS staining. The results demonstrated a significant increase in calcific nodules following si-p75NTR treatment, while the opposite trend was observed for oe-p75NTR (Fig. 3A). These preliminary findings suggest that p75NTR influences osteogenesis. Furthermore, we examined the expression of osteogenic-related genes, including Runx2, osteopontin, DLX5, osteocalcin, collagen type I, and BMP2, which are known markers for detecting cell osteogenic differentiation [31]. The results indicated significant upregulation of these markers after si-p75NTR treatment, whereas the opposite trend was observed for oe-p75NTR (Fig. 3B and C), further confirming the inhibitory role of p75NTR in BMSC osteogenic differentiation. Additionally, we assessed the expression levels of p75NTR and sortilin proteins in differentiating osteoblasts, and the results showed high expression of p75NTR and sortilin in osteoblasts. After treatment using si-p75NTR, the fluorescence intensity of p75NTR and sortilin was found to significantly decrease, and opposite results were observed for oe-p75NTR (Fig. 3D). This result further confirms that p75NTR can regulate the expression of sortilin in BMSCs, based on which we infer that p75NTR inhibits the osteogenic differentiation of rat BMSCs by regulating sortilin expression.

### Cellular experiments verify that si-p75NTR-BMSCs promote angiogenesis through sortilin

**Fig. 4:**
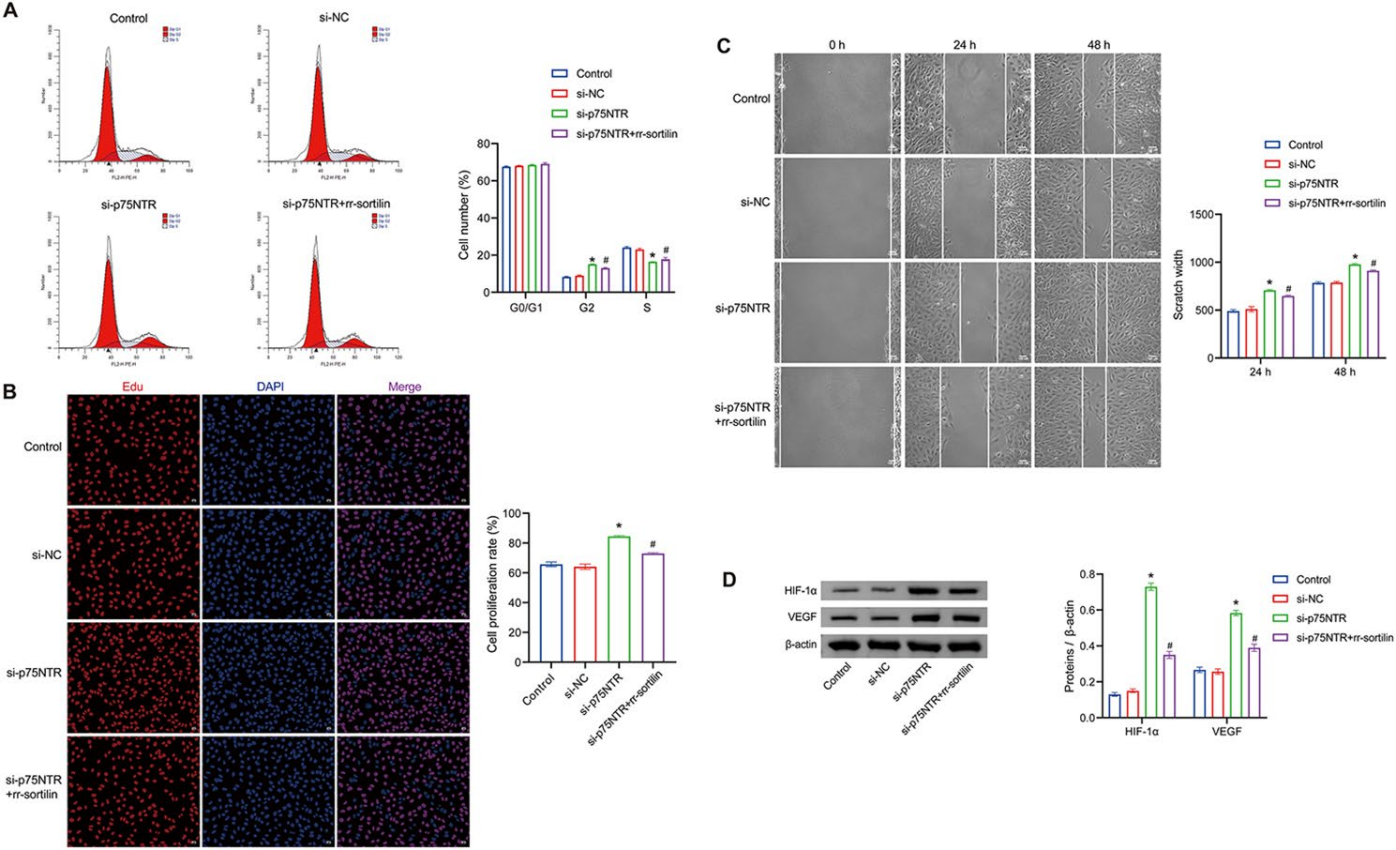
Cellular experiments verify that si-p75NTR-BMSCs promote angiogenesis through sortilin. (**A**) FCM analysis of HUVEC cell cycle. (**B**) EDU staining for detecting cell proliferation in HUVEC (Magnification: ×200, scale bar = 50 μm). (**C**) Cell scratch assay to evaluate the migratory ability of HUVECs. (**D**) WB analysis to measure the expression levels of HIF-1α and VEGF. *P < 0.05 vs. si-NC. #*P* < 0.05 vs. si-p75NTR

Next, we investigated whether p75NTR mediates the regulation of angiogenesis by BMSCs. Using a co-culture of BMSCs with HUVECs, we assessed the cell cycle of HUVECs and observed that most cells were in the G0/G1 phase, with no significant alterations induced by treatment with si-p75NTR or si-p75NTR + rr-sortilin. However, transfection with si-p75NTR led to a substantial increase in the number of cells in the G2 phase, which was partially weakened by si-p75NTR + rr-sortilin, resulting in a significant decrease in the number of cells in the G2 phase. Furthermore, si-p75NTR transfection significantly decreased the number of cells in the S phase, with si-p75NTR + rr-sortilin partially mitigating this effect (Fig. 4A). The proliferation results of HUVECs showed that si-p75NTR transfection significantly increased the cell proliferation rate, and this effect was weakened by si-p75NTR + rr-sortilin (Fig. 4B), aligning with the cell cycle findings. We also evaluated the migratory ability of HUVECs and found that si-p75NTR transfection significantly decreased the cell scratch width, indicating enhanced cell migration, which could be attenuated by si-p75NTR + rr-sortilin (Fig. 4C). Additionally, we examined the protein expression levels related to angiogenesis and observed that si-p75NTR transfection significantly increased the expression of HIF-1α and VEGF, while si-p75NTR + rr-sortilin weakened this effect (Fig. 4D). Based on these results, we hypothesized that silencing p75NTR could enhance angiogenesis by BMSCs through the regulation of sortilin.

### The si-p75NTR-BMSCs cells improve vascular regeneration and post-fracture healing in rats

**Fig. 5:**
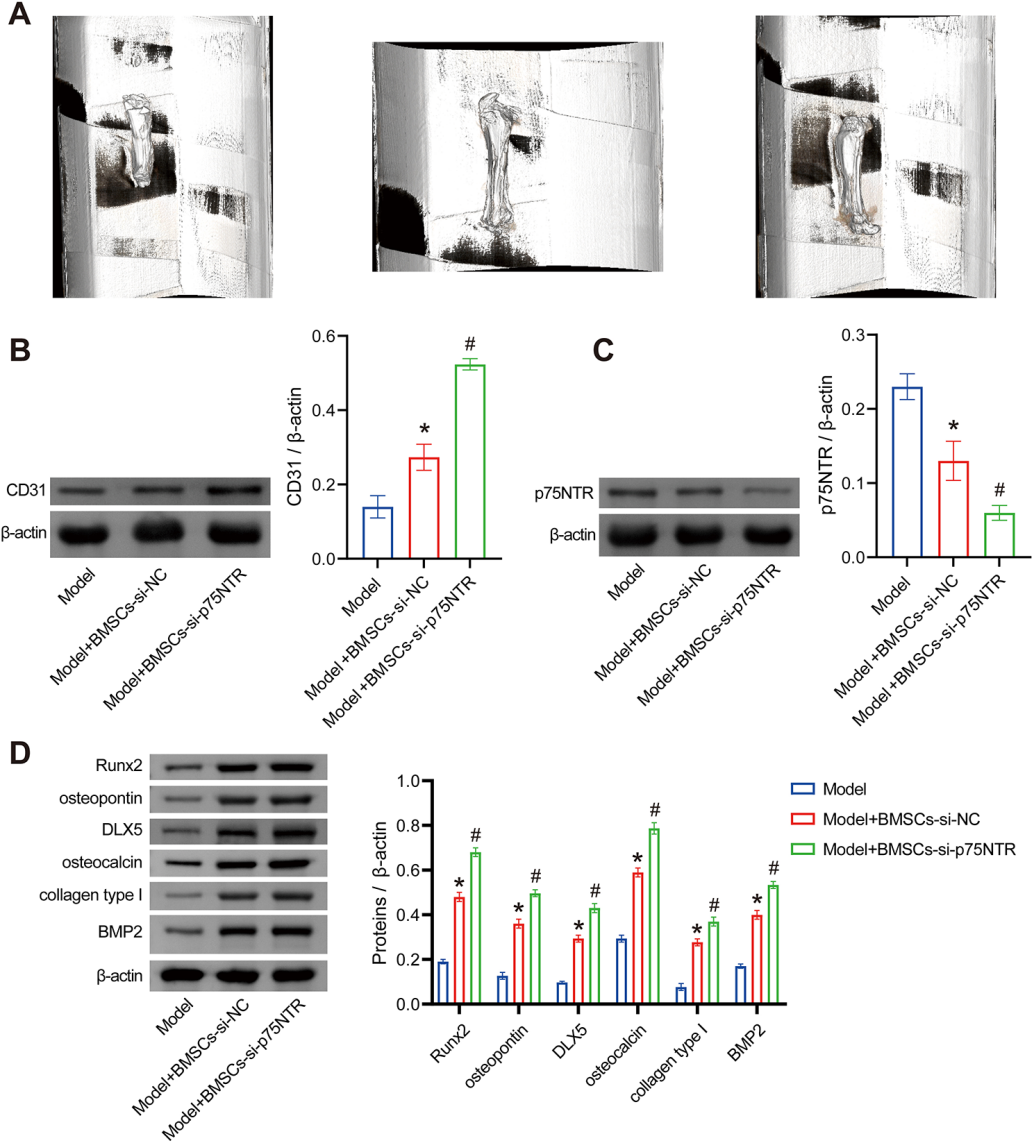
The si-p75NTR-BMSCs cells improve vascular regeneration and post-fracture healing in rats. (**A**) Micro-CT imaging shows the condition of rat femoral fractures. (**B**) WB analysis to measure the expression of CD31 in the healing tissue of the fracture. (**C**) WB analysis to measure the expression of p75NTR in the healing tissue of the fracture. (**D**) WB analysis to measure the expression of Runx2, osteopontin, DLX5, osteocalcin, collagen type I, and BMP2 in the healing tissue of the fracture. *P < 0.05 vs. Model. #*P* < 0.05 vs. Model + BMSCs + si-NC

First, we assessed the rats’ femoral fractures through CT imaging and observed that, compared to the Model group, the severity of fractures improved in the Model + BMSCs-si-NC group. The fractures in the Model + BMSCs-si-p75NTR group exhibited significantly better healing compared to the Model + BMSCs-si-NC group (Fig. 5A), which is consistent with our in vitro cell experiments. Then, we measured the expression levels of platelet endothelial cell adhesion molecule 1 (CD31) in the healed fracture tissues, and the results demonstrated that, compared to the Model group, CD31 expression increased in the Model + BMSCs-si-NC group and was significantly higher in the Model + BMSCs-si-p75NTR group compared to the Model + BMSCs-si-NC group (Fig. 5B). These findings suggest that si-p75NTR-BMSCs could promote angiogenesis. WB results revealed that, compared to the Model group, the expression of p75NTR significantly decreased in the Model + BMSCs-si-NC group, with a further substantial reduction in the Model + BMSCs-si-p75NTR group compared to the Model + BMSCs-si-NC group (Fig. 5C), indicating high p75NTR expression in fracture-healing tissue. Additionally, relative to the Model group, the Model + BMSCs-si-NC group showed increased expression of osteogenic-related proteins (Runx2, osteopontin, DLX5, osteocalcin, collagen type I, and BMP2). In the Model + BMSCs-si-p75NTR group, these proteins were also significantly elevated compared to the Model + BMSCs-si-NC group (Fig. 5D). Overall, these results support that p75NTR influences bone formation in rats with fractures and that silencing p75NTR may enhance vascular regeneration and bone healing in rats with fractures, potentially mediated by its regulation of rat BMSCs.

## Discussion

The human skeleton plays a crucial role in maintaining overall body stability [10]. Fractures can disrupt blood vessels in bones and the surrounding soft tissues [10]. Fracture healing is a complex restorative process, and delayed or non-union of fractures can occur in certain cases [32, 33]. Chun et al. demonstrated that stem cell therapy can effectively promote fracture healing but could be primarily complicated by a risk of infection [34]. Among stem cell types, BMSCs exhibit strong osteogenic potential and have been shown to facilitate both osteogenesis and vascular regeneration, ultimately contributing to bone repair [33, 35]. Nonetheless, the precise mechanisms underlying how BMSCs promote bone formation remain incompletely understood. It has been reported that p75NTR is widely distributed in tissues such as skeletal muscle and influences the survival of BMSCs [24, 36]. However, the specific role of p75NTR-mediated BMSCs in bone healing remains unclear. In this study, we initially isolated rat BMSCs and assessed their characterization, and also explored the influence of p75NTR on bone healing through a combination of in vitro and in vivo experiments, with the aim of determining the underlying mechanisms.

Sortilin, encoded by the SORT1 gene, is a type I transmembrane protein [37]. Elizabeth et al. have reported that p75NTR upregulates sortilin expression [38]. In our present study, we performed si-p75NTR transfections into BMSCs and observed a decrease in sortilin expression following p75NTR silencing. Additionally, CO-IP results indicated an interaction between p75NTR and sortilin. Moreover, our findings revealed that silencing p75NTR promoted apoptosis in BMSCs. Considering the report by Simona et al., which suggests that the upregulation of the p75NTR/sortilin receptor complex expression induces neuronal apoptosis [39], we hypothesize that p75NTR may promote apoptosis in BMSCs through sortilin.

Christa et al. discovered that p75NTR exhibits a suppressive effect on osteogenic differentiation in mice [40]. The osteogenic differentiation of BMSCs has been reported to be regulated by multiple transcription factors, including Runx2, osteopontin, and DLX5 [41]. Herein, our results showed that silencing p75NTR in BMSCs increased the expression of these regulatory factors. ARS staining results also demonstrated an increase in mineralized nodules upon silencing p75NTR. These preliminary findings suggest that silencing p75NTR could mediate the osteogenic differentiation of BMSCs. We further validated our findings through in vitro experiments. In contrast to the experiment conducted by Christa et al. [40], we established a rat fracture healing model by injecting engineered si-p75NTR-BMSCs cells into them. Our results demonstrated high expression levels of p75NTR in the tissues of the healing fractures. After silencing p75NTR, the rat fracture healing process was found to accelerate, and the expression of osteogenesis-related proteins in the fracture-healing tissue increased. These findings further support the idea that p75NTR plays a role in fracture healing. Moreover, we observed high expression levels of both p75NTR and sortilin in differentiated BMSCs, and p75NTR was found to positively regulate the expression of sortilin. Therefore, we hypothesize that p75NTR may mediate BMSCs and improve fracture healing by regulating sortilin.

As bone is a highly vascularized tissue, angiogenesis plays a crucial role in bone defect healing [42]. An intricate connection exists between angiogenic cells and osteogenic cells [42]. Key regulators of angiogenesis include VEGF and HIF-1α [8, 43]. Andrea et al. reported that p75NTR activation impairs endothelial function and angiogenesis [44]. In contrast to Andrea’s experiment, we conducted co-cultures of HUVECs and BMSCs to investigate whether si-p75NTR could mediate the effect of BMSCs on angiogenesis. Our results revealed that silencing p75NTR led to increased HUVEC cells, enhanced cell migration ability, and increased expression of VEGF and HIF-1α. These findings suggest that silencing p75NTR may mediate BMSCs to promote angiogenesis, which was then validated in subsequent experiments. It has been reported that Platelet/endothelial cell adhesion molecule-1 (PECAM-1), also known as CD31, promotes angiogenesis [45]. Therefore, we measured the expression of CD31 in the fracture-healing tissue of rats in our fracture-healing model and observed a significant increase in CD31 expression upon silencing p75NTR. Additionally, an improvement in fracture healing was observed in rats with silenced p75NTR. Furthermore, the pro-angiogenic effect of silencing p75NTR was weakened in BMSCs treated with rr-sortilin. Sortilin has been reported to play a significant role in lipid metabolism and vascular calcification in the literature [46]. Based on these findings, we hypothesize that si-p75NTR-BMSCs may promote angiogenesis and improve the healing of fractures by regulating sortilin expression.

### Limitations

This study focused on exploring the impact of silencing p75NTR in BMSCs on osteogenic differentiation and the therapeutic effects in vivo. In addition, although we provided preliminary evidence that silencing p75NTR in BMSCs might regulate angiogenesis through sortilin and confirmed that silencing p75NTR in BMSCs promotes angiogenesis following increased CD31 expression, further instigations are needed to clarify the specific mechanism via which sortilin is involved in the therapeutic effects of silencing p75NTR in BMSCs. This is a limitation of this study and also an area that needs further investigation in future studies.

## Conclusion

In conclusion, our study demonstrates that silencing p75NTR in rat BMSCs can enhance angiogenesis and accelerate fracture healing in rats by modulating sortilin expression. These findings open up new possibilities for treating non-union fractures and bone regeneration, providing a potential avenue for further research and therapeutic development in this field.

## Data Availability

The datasets used and analyzed during the current study are available from the corresponding author on reasonable request.
